# Key Determinants of Immune-Mediated Adverse Reactions to Oncology Drugs

**DOI:** 10.3390/cancers15235622

**Published:** 2023-11-28

**Authors:** Yihan Zhou, Shan Ding

**Affiliations:** 1 Medical Sciences Division, Department of Oncology, University of Oxford, Old Road Campus Building, Roosevelt Drive, Oxford OX3 7DQ, UK; 2 Department of Life Science, Imperial College London, South Kensington Campus, Exhibition Road, London SW7 2AZ, UK; shan.ding21@imperial.ac.uk

**Keywords:** cancer immunotherapy, immune checkpoint inhibitors (ICIs), immune-mediated adverse drug events (imADEs), immune-mediated adverse drug reactions (imADRs)

## Abstract

**Simple Summary:**

Immune-mediated adverse reactions (imADRs) are complex and arise from immune checkpoint inhibitors (ICIs). In particular, ICI antibody targets, combination therapy, the nature of tumor histology and burden, and personalized characteristics of patients affect the incidence of imADRs in clinical settings. In this review, we explore how these determinants correlate with the clinical incidence of imADRs, and suggest that it could be better managed by introducing appropriate biomarkers. Personalized treatments, such as choosing specific ICIs based on patient conditions, are also recommended. In conclusion, while imADRs have various determinants, conclusive evidence is still sought, emphasizing the need for further research.

**Abstract:**

To overcome the epidemiological severity of cancer, developing effective treatments is urgently required. In response, immune checkpoint inhibitors (ICIs) have been revealed as a promising resolution for treatment-resistant cancers across the world. Yet, they have both advantages and disadvantages, bringing therapeutic benefits while simultaneously inducing toxicity, and in particular, immune-mediated adverse drug reactions (imADRs), to the human body. These imADRs can be pathogenic and sometimes lethal, hampering health prediction and monitoring following the provision of ICI treatment. Therefore, it is necessary to collectively identify the determinant factors that contribute to these imADRs induced by ICIs. This article evaluated treatment-, tumor-, and patient-related determinants, and indicated a research gap for future investigations on the pathogenic mechanism of imADRs and translational conversion of determinants into clinical biomarkers to aid pharmacovigilance and cancer therapies.

## 1. Introduction

Cancer remains a worldwide healthcare problem, with approximately 19,300,000 new cases and 10,000,000 deaths in 2020 [1]. Apart from conventional therapy, including surgery, radiotherapy, and chemotherapy, there are challenges with resistance or tumor relapse [2]; the emergence of immunotherapy has revolutionized the development of cancer treatments and proposed a potent therapeutic approach that can target multiple cancer types [3]. The discovery of immune checkpoint inhibitors (ICIs), the most well-known and studied immunotherapeutic strategy for the negative regulation of immune inhibition, was awarded the Nobel Prize in 2018 for its novelty and colossal breakthrough. At present, ICIs entail both cytotoxic T-lymphocyte-associated antigen-4 (CTLA-4) inhibitors such as ipilimumab and programmed cell death receptor-1 (PD-1)/programmed cell death receptor-ligand 1 (PD-L1) inhibitors, including pembrolizumab, nivolumab, and durvalumab [4].

**Figure 1:**
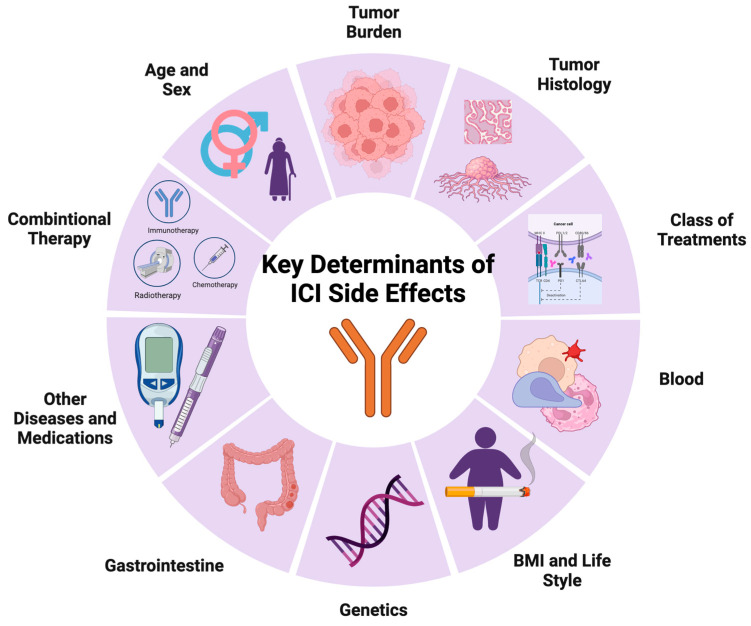
Visual overview of factors influencing immune-mediated adverse responses to oncologic therapies.

Despite the promise that ICIs hold as an anti-cancer therapeutic approach, several immune-mediated adverse drug events (imADEs) may also emerge simultaneously with the clinical benefits offered by ICIs. The causes of imADEs can vary, but only those proven to have a causal relationship with medicinal treatment are termed immune-mediated adverse drug reactions (imADRs) [5]. Identifying imADRs is a challenging but essential process in pharmacovigilance to increase the understanding of mechanisms and profiling of drug toxicity [6]. Particularly when pursuing anti-cancer efficacy to meet the therapeutic demand, the rapid expansion of the ICI market calls for an equivalent focus on the significance of safety concerns [7]. In response to leveraging the use of ICIs for therapeutic purposes, it is crucial to evaluate and identify the determinant factors of imADRs to aid measures taken to predict the incidence of imADEs, balance benefits and risks in treatment decisions, and potentially avoid future occurrences. Thus, this article will first demonstrate the general clinical incidence of imADEs and evaluate the determinants of imADRs across three aspects: treatment-, tumor-, and patient-related (Figure 1). The risk of imADRs associated with these determinants is summarized in Table 1. It will ultimately conclude that imADRs are complex and consequential of multiple determinants, to be validated as prospective biomarkers in the clinic.

## 2. Immune-Mediated Toxicity as a Cause of Death

In general, imADEs refer to the autoimmune condition in patients after receiving ICI treatment [29,30], and can manifest as hypophysitis, colitis, pneumonitis, thyroiditis, and myocarditis, exceedingly leading to death [3]. Specifically, the incidence of imADEs reached as high as 65.6% in phase III melanoma patients (ClinicalTrials.gov number: NCT00094653) receiving anti-CTLA-4 ipilimumab (three weekly doses of 3 mg/kg). Meanwhile, up to 14.5% were classified as grade ≥3 severity as defined in the National Cancer Institute’s Common Terminology Criteria for Adverse Events (CTCAE), and seven deaths were related to imADEs [31]. A higher dose (three weekly doses of 10 mg/kg) of ipilimumab in another phase III melanoma trial (ClinicalTrials.gov number: NCT00636168) increased the events classified as grade ≥3 severity to 41.6% and led to five imADE-related deaths [32].

In comparison, the imADEs of anti-PD-1 pembrolizumab are less common, but any-grade hypothyroidism, hyperthyroidism or pneumonitis was individually observed in 4 to 8% of non-small cell lung cancer (NSCLC) patients treated in a phase II/III study (ClinicalTrials.gov number: NCT01905657) with either 2 or 10 mg/kg of pembrolizumab three times each week, with three deaths due to pneumonitis [33]. In addition, 14.7% of phase III melanoma patients (ClinicalTrials.gov number: NCT02362594) were observed with any-grade imADEs following treatment with 200 mg/kg pembrolizumab three times each week, with 7% belonging to grade ≥3 CTCAE severity and one patient dying due to myositis [34]. Nivolumab also led to any-grade drug-related ADEs in 74.3% of melanoma patients free of mutations at *BRAF* oncogenes, in which pruritus and rash accounted for 17 and 15% of symptoms, respectively, in a phase III trial (ClinicalTrials.gov number: NCT01721772) [35].

By contrast, anti-PD-L1 treatment is even less toxic than anti-PD-1 treatment for the preservation of PD-1/PD-L2 immune signaling that maintains homeostasis. For instance, Khunger et al. [36] reported that the incidence of any-grade pneumonitis was significantly higher at 3.6% (versus 1.3% with PD-L1 inhibitors, *p* < 0.01) for PD-1 inhibitors and that of grade ≥3 pneumonitis was 1.1% (versus 0.4% with PD-L1 inhibitors, *p* < 0.05) of NSCLC patients. Another systemic analysis similarly presented that 16% (versus 14% with PD-L1 inhibitors) of NSCLC patients receiving PD-1 inhibitors experienced imADEs, and 4% (versus 2% with PD-L1 inhibitors, *p* < 0.05) developed pneumonitis [37].

## 3. Key Determinants of imADRs in ICI Treatment

### 3.1. Treatment-Related

#### 3.1.1. Class of ICI Treatment

The class of ICIs, as the cause of imADRs, can help elucidate the severity and manifestation of these reactions that patients are more likely to encounter following treatment. Pertaining to the imADE incidence described previously, it is apparent that anti-CTLA4 antibodies may render more frequent and severe imADRs than anti-PD-1 or anti-PD-L1 antibodies. This difference in imADE profiling can be explained by the distinct places and timing of their regulatory actions on immunity. By contrast, CTLA-4 is inhibited in the initial stages when naïve T cells are activated in the lymph node in comparison to PD-1/PD-L1 inhibition, which occurs at a later stage of anti-tumor effector T cell responses in the peripheral tissues [38]. In addition, such distinction in immune regulatory mechanism corresponds to different toxicity profiles in mice models, where CTLA-4 deficiency led to lethal lymphoproliferative disorders [39] and PD-1 knockout broke down the peripheral self-tolerance, giving rise to less severe arthritis, glomerulonephritis, and humoral immunoglobulin G3 (IgG3) deposits [40].

Additionally, imADRs vary in their actual manifestation in an organ-specific pattern. These may take place when immune checkpoints are expressed in certain organs other than the therapeutic targets. Typically, hypophysitis (odd ratio (OR) 6.5, *p* < 0.0001) was an endocrine imADRs commonly seen in patients after receiving ipilimumab rather than PD-1/PD-L1 inhibition treatment [8], potentially because of complement activation and phagocytosis triggered by the inhibition of CTLA-4 molecules ectopically expressed by pituitary cells [41,42]. Likewise, the utilization of anti-PD-L1 antibodies tended to induce renal imADRs as PD-L1 expression was also discovered on the renal tubular epithelium [9,10]. On the other hand, the finding that hypothyroidism (OR 4.3, *p* < 0.0001) was a more common imADR recorded in anti-PD-1 treatment [8] may be explained by the general humoral immune enhancement upon treatment, including the side effect of surging levels of pre-existing anti-thyroid antibodies above the detectable baseline [43].

#### 3.1.2. Combined Anti-Cancer Treatments

Most of the time, patients are subjected to not only one anti-cancer drug or therapy but rather a combined treatment that can expose them to greater toxicity. In cases where more than one ICI is given to patients, the profiling of imADRs can be further augmented. The combination of ipilimumab and nivolumab resulted in a higher incidence of imADRs in 54% (versus 24% with ipilimumab monotherapy) of phase I BRAF-mutated melanoma patients (ClinicalTrials.gov number: NCT01927419) [12]. Amongst them, 23.4% (versus 13.0% with ipilimumab monotherapy) were diagnosed with colitis, 10.6% (versus 4.3% with ipilimumab monotherapy) with pneumonitis and 11.7% (versus 6.5% with ipilimumab monotherapy) with hypophysitis at any grade of CTCAE severity. The imADR profiling may also be affected by the previous introduction of other types of immunotherapy, such as cancer vaccines [44] and adoptive therapy [45], that can result in the diversification of epitope specificity or so-called epitope spreading [13]. The presence of epitope spreading can be beneficial to triggering and promoting effective anti-cancer responses but may simultaneously increase the risk of developing imADRs.

Radiotherapy uses high-energy radiation to kill cancer cells, yet also causes non-specific damage to the surrounding non-cancerous tissue, leading to an immune attack against self-antigens released by the damaged cells. Shaverdian et al. [46], in a phase I NSCLC trial (ClinicalTrials.gov number: NCT01295827), found that, following pembrolizumab administration, 8% of patients previously treated with thoracic radiotherapy were observed with treatment-related pneumonitis and 4% belonged to grade ≥3 CTCAE severity, compared to 1% of those that were not. The any-grade immune toxicity had greater, yet mostly mild, incidence in 39% of patients when radiotherapy was administrated within two weeks of ICI administration, compared to 23% of other patients [16].

Similarly, higher risk also occurs when ICI treatments are combined with chemotherapy. Despite the increase in overall survival outcomes in a phase III NSCLC trial (ClinicalTrials.gov number: NCT02775435), any-grade immune-mediated toxicity occurred in 28.8% (versus 8.6% with placebo plus chemotherapy) of patients subjected to pembrolizumab plus chemotherapy treatment, with 10.8% (versus 3.2% with placebo plus chemotherapy) being grade ≥3 severity [17]. A similar observation was also made by Antonia et al. [47] that immune-mediated toxicity presented at any grade in 24.2% (versus 8.1% with placebo plus chemotherapy) of phase III NSCLC patients (ClinicalTrials.gov number: NCT02125461) subjected to both durvalumab and chemotherapy, and 3.4% (versus 2.6% with placebo plus chemotherapy) were grade ≥3 severe. Moreover, a meta-analysis reported the significance of ICI addition to chemotherapy in increasing the risk of pneumonitis (OR 2.67, *p* < 0.00001) [48]. Interestingly, reduced grade 3 or 4 side effects were observed in metronomic vinorelbine and atezolizumab combinational therapy (13.3%) [49], compared to atezolizumab monotherapy (30.1%) [50], in NSCLC. The low-risk profile was also shown in the metronomic administration of capecitabine for sorafenib-resistant HCC [51].

Some clinical trials that investigated the effect of combining tyrosine kinase inhibitor (TKI) and ICI on treating RCC (renal cell carcinoma) [52], NSCLC [53], hepatocellular carcinoma [54], and various solid tumors [55] have shown promising outcomes, yet raised concerns towards imADRs. In metastatic RCC, patients receiving a list of ICIs, TKIs and their combinations (ClinicalTrials.gov number: NCT03793166) had both objective response rate and median progression-free survival (mPFS) rate decreased, but toxicities increased throughout successive lines of treatment. Although feasible as a therapy in first-line settings, 52% of patients receiving second-line treatments and beyond developed grade ≥3 adverse events such as diarrhea/colitis, fatigue and more. While the single-arm trial of the combination of lenvatinib and pembrolizumab in hepatocellular carcinoma (ClinicalTrials.gov number: NCT03006926) reported 67% of patients developing grade ≥3 treatment-related toxicities, the IMBRAVE 150 trial (ClinicalTrials.gov number: NCT03434379) showed a lower incidence of grade 3 or 4 severity, at 15.2%, in TKI/ICI administration. However, serious side effects occurred at a higher rate in patients treated with atezolizumab–bevacizumab (38.0%) than those receiving sorafenib (30.8%). For advanced NSCLC, the use of ICIs in front-line therapy following resistance to TKIs has shown an increase in mPFS by an average of 3.8 months. However, the direct combination of an epidermal growth factor receptor (EGFR) downstream TKI with ICI therapy increased the prevalence of any-grade toxicities, particularly with severe effects on skin and interstitial lung diseases, leading to early termination of the CAURAL (ClinicalTrials.gov number: NCT02454933) and TATTON (ClinicalTrials.gov number: NCT02143466) trials.

### 3.2. Tumor-Related

#### 3.2.1. Tumor Histology

Numerous studies have demonstrated the association of imADR presentation with primary tumor histology [8,15,56]. Despite the scarcity of head-to-head studies, Khoja et al. [11] found that melanoma was mainly associated with colitis (OR 4.2, *p* < 0.001), pruritus (OR 2.4, *p* < 0.0001) and rashes (OR 1.8, *p* < 0.0001) compared to NSCLC that was more associated with pneumonitis (OR 2.3, *p* < 0.0001) following anti-PD-1 treatment. Meanwhile, RCC patients were more likely to develop pneumonitis (OR 2.9, *p* < 0.0001) compared to hypothyroidism (OR 3.6, *p* < 0.0001), pruritus (OR 1.5, *p* < 0.05) and rashes (OR 1.6, *p* < 0.05) developed in ICI-treated melanoma patients [8]. Furthermore, acute myeloid leukemia and myelodysplastic syndromes after ICI treatments were more associated with grade ≥3 immune-mediated toxicity, including 15% for dermal, 11% for hepatic, 4% for pneumonitis, and 4% for immune thrombocytopenia [56]. The onset of interstitial lung disease occurred in lung cancer patients mainly receiving anti-PD-1 antibodies in the median of 2.1 months, which was significantly (*p* < 0.05) earlier than the median of 5.2 months in melanoma patients [15]. Despite the fact that the clinicopathogenesis of imADRs has been under-examined, it can be implied that the variety of imADRs induced by the same ICI could be due to the inter-individual difference in tumor microenvironments and sensitive organs that react to ICI drugs and, subsequently, develop an imADR.

#### 3.2.2. Tumor Burden

Tumor burden can not only help determine the stage of disease progression and severity but also evaluate the vulnerability of patients to unwanted immune responses. For instance, 28.8% of melanoma or NSCLC patients with ≥two metastatic sites had at least one case of life-threatening immune-mediated toxicity following ICI treatment [16]. The severe immune side effect was also related to the tumor load ≥90 mm (OR 8.62, *p* < 0.01) after either anti-CTLA-4 or anti-PD-1 treatment [17], and the high occurrence of tumor mutations (Pearson correlation (r) 0.704, *p* < 0.001) after anti-PD-1 treatment [57].

### 3.3. Patient-Related

#### 3.3.1. Demographics

Although the significant association between the risk of imADRs and demographic factors, such as age and gender, was poorly established, Asada et al. [21] reported by using the US Food and Drug Administration (FDA) Adverse Event Reporting System (FAERS) database, that patients aged ≥60 were more resistant to developing ICI-related pneumonitis (OR 0.68, *p* < 0.001) than those aged <60. In addition, a retrospective study that included melanoma, NSCLC, and RCC patients treated with ICIs discovered no difference in imADEs among age groups, but endocrine toxicity was more related to patients aged <65 and dermal toxicity to those aged ≥75 [58]. Betof et al. [22] provided additional evidence of a significantly higher incidence of arthritis in melanoma patients aged ≥65 and <75 at 10.8% (*p* < 0.05) after PD-1/PD-L1 inhibition treatment.

In addition to age, gender presents an organ-specific imADR profiling that may be due to an unknown interaction with sex hormones. For instance, females had an increased risk of ipilimumab-mediated toxicity (OR 1.5, *p* < 0.05) than male melanoma, NSCLC and RCC patients [59], yet were less susceptible to cardiac imADRs, including arrhythmias (OR 0.81, *p* < 0.001), coronary artery disease (OR 0.63, *p* < 0.001), myocardial infarction (OR 0.60, *p* < 0.001), myocarditis (OR 0.59, *p* < 0.001) and pericarditis (OR 0.50, *p* < 0.001) [20]. Triggianese et al. [60] also summarized the male dominancy in neurologic, thyroidal, and dermal imADR incidence.

#### 3.3.2. Physiological Parameters and Lifestyle

Demographics alone are inadequate to predict imADR toxicity following ICI treatments. The information on physiological parameters and lifestyle supplemented the confidence in managing ICI-related imADRs. Perceiving the risks associated with high body mass index (BMI), ≥25 kg/m^2^, after pembrolizumab treatment for lung cancer, melanoma, lymphoma, gastric cancer, urothelial and other cancers is straightforward (OR 1.08, *p* < 0.05) [21], as obesity is related to upregulated markers of chronic inflammation [61]. The poor performance status, defined as grade ≥2 according to the Eastern Cooperative Oncology Group (ECOG) standard, and smoking history of ≥50 packs/year elevated the incidence of ICI-related interstitial lung disease in lung cancer patients [22]. Low muscle attenuation could lead to increased high-grade ipilimumab-related toxicity (OR 3.57, *p* < 0.05) and the incidence of colitis of 16.7% (versus 2.1% with high muscle attenuation, *p* < 0.05) in melanoma patients [62].

#### 3.3.3. Genetic Predisposition

Genetic predisposition to the human leukocyte antigen (HLA) is always considered a confounding factor contributing to autoimmunity and imADRs. HLA is the surface peptide that binds to and presents antigens to T cells to activate the adaptive system [63]. Ali et al. [23] performed a prospective observational study in which ICI-treated melanoma or NSCLC patients with HLA-DRB1*11:01 allele were significantly associated with higher risk of developing pruritus (OR 4.53, *p* < 0.01) and HLA-DQB1*03:01 allele with increased risk of colitis (OR 3.94, *p* < 0.05). Moreover, HLA-DR4, HLA-B52 and HLA-Cw12 in the Japanese database were more dominant in 81.8% (versus 33.5% of healthy patients, *p* < 0.01), 63.6% (versus 21.0% of healthy patients, *p* < 0.01) and 70% (versus 21.3% of healthy patients, *p* < 0.01), respectively, of ICI-treated melanoma, NSCLC or gastric cancer patients observed with pituitary immune-mediated toxicity [64]. Inflammatory arthritis induced by ICI treatment was more associated with HLA-DRB1*04:05 genotyping (OR 8.6, *p* < 0.05) [65], whilst HLA-DPA1*02:02 (*p* < 0.05) and HLA-DPB1*05:01 (*p* < 0.01) were enriched in ICI-induced type 1 diabetes mellitus [66].

In addition to the HLA genotype, the single nucleotide polymorphism (SNP) variants can also predispose patients to the risk of developing imADRs. The presence of the variant A allele in SNP rs11743438 mapped to the *gamma-aminobutyric acid type A subunit Pi* (*GABRP*) gene (OR 4.3, *p* < 0.00001), the variant A allele in SNP JHU_20.57183980 mapped to the *desmocollin 2* (*DSC2*) gene (OR 6.9, *p* < 0.00001), the variant G allele in SNP rs563284422 mapped to the *bromodomain adjacent to zinc finger domain 2B* (*BAZ2B*) gene (OR 4.2, *p* < 0.0001) and the variant T allele in SNP rs3026321 mapped to the *semaphorin 5A* (*SEMA5A*) gene (OR 19.8, *p* < 0.0001) could increase the risk of developing ICI-related toxicity in melanoma patients [27]. Marschner et al. [67] further argued that the SNP rs2910164 in the *microRNA-146a* (*MIR146A*) gene in ICI-treated melanoma or lung cancer patients was associated with the increased severity of imADRs, potentially attributed to the increased neutrophil count.

#### 3.3.4. Blood Characteristics

Biological characteristics, especially those inflammatory factors in circulation, can also affect potential exposure to imADRs. ICI-related toxicity was associated with blood cell indexes, including the absolute counts of lymphocytes (>2.6 k/μL, OR 4.30), monocytes (>0.29 k/μL, OR 2.34), platelets (>145 k/μL, OR 2.23) and the ratios of neutrophil to lymphocyte (≤5.3, OR 2.07), monocytes to lymphocytes (≤0.73, OR 2.96) and platelets to lymphocytes (≤534, OR 5.05) [25]. In a study assessing 34 cytokines in melanoma patients, Tyan et al. [68] demonstrated the association of ICI-related dermatitis to higher angiopoietin 1 (Ang-1) (*p* < 0.01) and CD40 ligand (CD40L) (*p* < 0.01) levels, pneumonitis to higher interleukin-17 (IL-17) (*p* < 0.01) levels and colitis to lower granulocyte-colony stimulating factor (GCSF) levels. The presence of pre-existing autoantibodies could aggravate the risk of ICI-related toxicity (OR 3.25, *p* < 0.01); for example, rheumatoid factors led to more frequent skin reactions and anti-thyroid autoantibodies led to thyroid dysfunction in NSCLC patients [69]. Other factors included high lactate dehydrogenase levels (≥245 U/L, OR 2.39, *p* < 0.05) in patients with lung, esophagus or gastrointestinal [70], as well as high albumin levels (≥3.6 g/dL, *p* < 0.05) in patients with melanoma, NSCLC, RCC or gastric cancer [71].

#### 3.3.5. Gastrointestinal Characteristics

From the gastrointestinal perspective, intestinal microbiota and stool are also associated with localized imADRs. The enrichment in *Faecalibacterium* and other Firmicutes could lead to a higher incidence of ipilimumab-related colitis in melanoma patients [72] and low *Bacteroidetes*/*Firmicutes* ratios presented in NSCLC, SCLC, or uterine endometrial dedifferentiated cancer patients with ICI-related acute pancreatitis [26], which is consistent with the discovery of protection of *Bacteroidetes* phylum from colitis in melanoma patients [73]. In addition, approximately 84.5% of melanoma, solid, or hematological cancer patients with ICI-related colitis were positive for decal lactoferrin and 56.4% were positive for calprotectin >150 mcg/g with a sensitivity of 90 and 68%, respectively, to detect histological inflammation [74].

#### 3.3.6. Other Diseases and Medications

A variety of research has correlated imADRs with organ dysfunctions, such as previous lung diseases that were prone to ICI-induced pneumonitis (OR 2.86, *p* < 0.01) [27]. Cardiovascular conditions were linked with heart immune-mediated toxicity [28] and a lower estimated glomerular filtration rate, specifically a decline of <30 mL/min per 1.73 m^2^, with acute kidney injury in melanoma, lung, or genitourinary cancer patients (OR 1.99) [75]. This corresponds to an elevated immune attack at the place of organ damage for repair, which unexpectedly introduces localized imADRs during ICI treatments. Patients with an autoimmune history (OR 2.57, *p* < 0.01) or even just a family history (OR 5.98, *p* < 0.01) are more prone to developing imADRs [25]. Similarly, Shimozaki et al. [71] suggested a relationship between type 1 hypersensitivity reaction and ICI-related toxicity in melanoma, NSCLC, RCC, or gastric cancer patients. However, other studies have contradictory findings of no difference between NSCLC patients with or without autoimmune disease [76], diabetes, cardiovascular disease, or chronic obstructive pulmonary disease [77]. Liver conditions, on the other hand, do not seem to correlate with the levels of adverse effects following ICI treatment. Specifically, the rate of treatment-related toxicities did not show significant differences when comparing the Child–Pugh B group with the Child–Pugh A group [78]. Moreover, it is uncommon for patients to develop post-ICI hepatotoxicity with pembrolizumab (0.7%), nivolumab (1.8%), atezolizumab (0.9–1.3%) and avelumab (0.9%) treatments [79]. Clinical and FDA reports have reaffirmed this low incidence rate: 5.3% of 151 hospital patients treated with ICIs developed grade ≥3 liver injury [80], and there were merely 654 cases of hepatic failure among 9,647,655 records in the US FAERS database [81].

To treat co-morbidities, patients need to take prior or concomitant medications for purposes other than treating cancer, including proton pump inhibitors (OR 2.85) [75], corticosteroids (OR 1.9, *p* < 0.05) [82], antibiotics (OR 2.12, *p* < 0.01) [83] or diuretics (OR 4.3, *p* < 0.001) [82] that could also induce imADRs in melanoma, lung or genitourinary cancer patients, as well as influenza vaccination [84]. Among them, medications such as proton pump inhibitors (PPI) were especially known as the cause of acute interstitial nephritis and thus kidney dysfunction [85], yet clinical results on their effect on imADR were conflicting. An Italian study of 360 patients demonstrated a 92% incidence of gastrointestinal toxicities for patients exposed to PPI prior to ICI therapy, compared to 8% of the control group [86]. Noticeably, they excluded non-gastrointestinal related imADR in their estimation; no comparison of total imADR was made between PPI-exposed and non-exposed groups. On the contrary, another clinical study showed no correlation between using PPI and imADR occurrence, including colitis, in ICI therapies [87]. Currently, the mechanism of IPP-induced imADR remains elusive due to limited clinical studies. Furthermore, the intake of vitamin D supplements could ameliorate inflammation and autoimmunity [88,89], though whether this knowledge can also be applied in the case of imADRs requires further investigations. 

## 4. Discussion

Overall, imADRs have been complicated with multiple determinants, and one significant factor is unlikely to determine the incidence of imADRs. Yet, there is no evidence supporting any proposed mechanism behind them, and the weight of each determinant has also been under investigation. From the perspective of cancer treatment, different classes of ICI drugs exhibit distinct imADR profiles due to their specific target location and timing. Moreover, the use of combined therapy, a common strategy for its synergistic efficacy, introduces additional danger to patients due to the concurrent presence of synergistic toxicity. Tumor histology and burden can also partly affect the imADR profiling of a single ICI and, more importantly, the patient’s personal characteristics, regardless of whether these are demographic, physiological, genetic, biological or medical, constitute a greater variation in predicting both outcomes and toxicity from ICI drugs. The advances in immunotherapy will soon evolve to be an indispensable cancer treatment in the short-term and, thereby, our knowledge of imADRs needs to be further refined, and the interaction of multiple determinants must be understood.

To combat such variation and help with the early detection and management of imADR management following ICI treatment, biomarkers should be introduced in clinical use and validated to quantify the risk of imADRs. Clinical trials should be designed to conclusively analyze and translate determinants into biomarkers instead of solely conducting meta-analyses or systematic reviews. Pre-treatment toxicity tests can be based on biomarker index to evaluate the imADR risk personalized to each patient to qualify the use of certain ICI drugs and provide advice for better health benefits. For instance, obese patients who are more susceptible to imADRs should be supplied with anti-PD-L1 antibodies as they have milder immune-mediated toxicity yet the same efficacy as anti-PD-1 antibodies [37]. Patients should not use anti-CTLA-4 antibodies when their microbiota have a low level of *Bacteroidetes*, and those with poor prognosis on genetic predisposition should attempt other types of cancer therapy instead. Combined therapy is essential but sufficient time intervals should allow patients to recover from the previous therapy before entering the next. Other medications that are known to cause damage and worsen imADRs should be swapped for less harmful alternatives.

Beyond cancer treatment, improving the understanding of imADRs can also facilitate the establishment of disease mouse models using immune checkpoints to mimic the autoimmune condition, which can provide insights into autoimmune pathogenesis. Despite these prospects, the approach to studying imADRs can be largely impeded by invasive access to biological specimens, as it may be difficult to obtain patients’ consent to take risks beyond therapeutic toxicity. Meanwhile, how to pursue the investigation of imADR clinicopathology with the greatest protection provided to patients is the next inevitable practical question in clinical ICI studies.

## 5. Conclusions

In summary, ICI-related imADRs are associated with several determinants but none of them are currently proven with reliable and conclusive support. Further investigations are warranted to assess the mechanism of how determinants individually and jointly contribute to imADR development and validate clinical biomarkers to hint at imADR prediction, early detection and management concerning the therapeutic use of ICIs.

## Figures and Tables

**Table 1:** Determinants and associated clinical incidents of immune checkpoint inhibitor-induced adverse reactions (imADRs).

Key Determinants		Clinical Incident
Treatment	Antibody target for ICI	imADR risk: anti-CTLA-4 > anti-PD-1 or anti-PD-L1 Risks of hypophysitis [8], renal imADR [9,10] and hypothyroidism [11]: higher in anti-PD-L1
	Combined anti-cancer treatments	Higher imADR incident in combining ipilimumab and nivolumab [12], preceding immunotherapy [13], radiotherapy [11] and chemotherapy [14]
Tumor	Histology	Anti-PD-1 treatment causes dermatological symptoms in melanoma but pneumonitis in NSCLC [11] Anti-PD-1 induce earlier onset of lung diseases in lung cancer [15]
	Burden	Higher tumor load and metastatic sites associated with severe side effects of anti-CTLA-4 or anti-PD-1 [16,17]
Patient	Demographics	ICI-related pneumonitis: younger > older [18] Arthritis after PD-1/PD-L1 inhibition: older > younger [19] Risk of ipilimumab-mediated toxicity: female > male [20]
	Physiological Parameter and Lifestyle	Pembrolizumab: obesity > healthy [21] Lung disease in lung cancer: ≥50 pack-year elevated the incidence [22]
	Genetic predisposition	Pruritus and colitis: higher risk in certain HLA genotype [23] SNP variants increase risk of ICI-related toxicity [24]
	Blood and gastrointestinal	Higher lymphocyte, monocyte, platelet, ratio immune cells associate with ICI-related toxicity [25] Ipilimumab-related colitis: higher risk with higher Firmicutes [26]
	Other diseases and medications	Higher risks associated with previous lung disease [27], cardiovascular conditions [28] or autoimmune history [25]

## Data Availability

No new data were created or analyzed in this study. Data sharing is not applicable to this article.
